# Recycled Materials and Lightweight Insulating Additions to Mixtures for 3D Concrete Printing

**DOI:** 10.3390/ma18184387

**Published:** 2025-09-19

**Authors:** Marcin Maroszek, Magdalena Rudziewicz, Karina Rusin-Żurek, Izabela Hager, Marek Hebda

**Affiliations:** 1 Department of Materials Engineering, Faculty of Materials Engineering and Physics, Cracow University of Technology, Warszawska 24, 31-155 Kraków, Poland; marcin.maroszek@doktorant.pk.edu.pl (M.M.); magdalena.rudziewicz@doktorant.pk.edu.pl (M.R.); karina.rusin-zurek@pk.edu.pl (K.R.-Ż.); 2 Chair of Building Materials Engineering, Faculty of Civil Engineering, Cracow University of Technology, Warszawska 24, 31-155 Kraków, Poland; izabela.hager@pk.edu.pl

**Keywords:** 3D concrete printing (3DCP), recycled aggregates, lightweight additives, insulating additives, sustainable construction, circular economy, carbon footprint, thermal conductivity

## Abstract

Three-dimensional concrete printing (3DCP) is advancing rapidly, yet its sustainable adoption requires alignment with circular-economy principles. This study evaluates the substitution of natural aggregates with recycled constituents, 3DCP waste, brick debris, glass cullet, mixed rubble, fly ash, and slag, and the use of lightweight fillers (expanded perlite, lightweight expanded clay aggregate (LECA), and expanded polystyrene (EPS)) to reduce density and improve insulation. Key properties, such as particle-size distribution, printability, mechanical performance, thermal conductivity, and water absorption, were determined. Results indicate that grading strongly affected mixture behavior. Narrow distributions (fly ash, milled 3DCP waste) enhanced extrudability, while broader gradings (glass, rubble, slag) increased water demand and extrusion risks. Despite these differences, all systems remained within the printable window: flow spread decreased with most recycled additions (lowest for brick) and increased with glass. Mechanical responses were composition-dependent. Flexural strength typically decreased. Compressive strength benefited from broader gradings, with replacement levels up to ~6% enhancing strength due to improved packing. Loading anisotropy typical of 3DCP was observed, with perpendicular compressive strength reaching up to 13% higher values than parallel loading. Lightweight fillers significantly reduced thermal conductivity. LECA provided the best compromise between strength and insulation, perlite showed intermediate behavior, and EPS achieved the lowest thermal conductivity but induced significant strength penalties due to weak matrix-EPS interfaces. Water absorption decreased in recycled-aggregate mixes, whereas lightweight systems, particularly with perlite, retained higher uptake. The results demonstrate that non-reactive recycled aggregates and lightweight insulating fillers can be successfully integrated into extrusion-based 3DCP without compromising printability.

## 1. Introduction

The construction sector is an energy-intensive and emissions-intensive branch of industry, within which the cement industry remains the key contributor. Emission sources in this sector are distributed as follows: approximately 60% of total emissions are process emissions resulting from limestone calcination (thermal decomposition of CaCO_3_ to CaO, inevitably accompanied by CO_2_ release) [1]. The remaining 40% originates mainly from fuel combustion required to achieve kiln operating temperatures. In addition, indirect emissions associated with electricity consumption account for about 6% of total CO_2_ output; consequently, fuel combustion contributes ~34% and electricity ~6% of total emissions [2,3,4].

Recent analyses indicate that in 2023, the environmental footprint of the EU-27 cement sector was characterized by a production volume of 161.1 Mt and a consumption level of 150.8 Mt (≈336 kg per capita), in comparison to a global output of approximately 4.03 Gt [5]. In extra-EU trade, cement and clinker imports to the Union totaled 9.3 Mt, with Turkey (35.8%), Algeria (19.2%), and Ukraine (12.6%) representing the principal sources [6]. Current emission-intensity indicators for the EU-27 (direct emissions: fuel + process; excluding electricity) indicate ~0.58 t CO_2_ per t of cement products (2022) and ~0.81–0.84 t CO_2_ per t of clinker (2022) [5]. Applying these intensities to 2023 production yields on the order of ~93.9 Mt CO_2_ (161.1 Mt × 0.583 t CO_2_/t) for cement and ~99–103 Mt CO_2_ attributable to the clinker share in that cement, under a conservative assumption of a clinker-to-cement ratio of 0.758 (EU average, 2022; without adjusting for clinker trade) [7,8].

In response to these conditions and regulatory pressure, the cement industry increasingly substitutes conventional raw materials—primarily limestone—with secondary materials derived from industrial and post-consumer waste streams. These inputs supply the requisite oxides (CaO, SiO_2_, Al_2_O_3_, Fe_2_O_3_) and include, among others, fly ash (FA) from alternative-fuel combustion, metallurgical slags, dewatered sewage sludge, fine recycled aggregates from crushed concrete, and selected fractions of construction and demolition waste (CDW) [9,10,11]. A key advantage is their often pre-decarbonated nature, which can substantially reduce process-related CO_2_ emissions during clinker manufacture.

Within the circular-economy paradigm, interest is growing not only in valorizing industrial by-products but also in incorporating CDW fractions into formulations intended for three-dimensional concrete printing (3D concrete printing, 3DCP). Recycled components in this group include, inter alia, recycled fine aggregate (RFA), ground brick waste (GBW), recycled concrete aggregate (RCA), and recycled clay brick waste (RCBW). Numerous studies have demonstrated their applicability as partial or complete substitutes for natural aggregates [12]. With appropriate preparation, comprising crushing, screening, and, where necessary, surface modification, demolition-derived fractions can replace aggregates without compromising the key mechanical and rheological parameters required in additive manufacturing systems [13]. In practice, successful implementation requires not only feedstock preparation but also control of fines content, cleanliness (removal of contaminants and adhered mortar), and shaping of the grading curve to limit segregation and ensure stable layer-by-layer deposition [13]. GBW deserves particular attention because it combines a filler effect with pozzolanic activity; this material can support strength development and durability of printed elements while diverting waste from landfills [12].

The importance of microstructure and particle geometry in 3DCP mix design has been emphasized by Ivanova and Mechtcherine: particle-size distribution, specific surface area, and grain morphology significantly govern yield stress, extrudability, and interlayer stability—parameters that determine the success of layer-by-layer deposition. This highlights the necessity of precise adjustment of particle texture and morphology, e.g., surface roughness, which generally enhances thixotropy and improves shape retention upon nozzle extrusion. On the other hand, an excess of very fine fractions increases water demand and the risk of blockages within the feed and delivery system [14,15]. Accordingly, a printable mix design must balance fresh-state properties with a controlled rate of structural build-up over time.

Further experimental studies showed that incorporating RCA into fresh mixtures leads to time-dependent improvements in rheology. The yield stress of RCA-bearing mixtures increased exponentially, whereas the shear modulus rose linearly during the first 15 min after mixing. Notably, buildability, the ability of a mixture to maintain geometric stability during multi-layer printing, increased proportionally with RCA content. The observed trend can be attributed to RCA-induced modification of the water–binder equilibrium and accelerated structural build-up (SBU), which improves post-deposition filament stability. However, it also necessitates tighter control of superplasticizer dosage and mixing parameters to maintain extrusion continuity and avoid excessive flow resistance [16,17]. Moreover, partial replacement (30 wt%) of ordinary Portland cement (OPC) with recycled fines (<75 µm) sourced from RFA and RCBW has yielded cement pastes with rheological and mechanical properties suitable for additive manufacturing applications [18]. Incorporation of RFA into mortars significantly enhanced buildability: all tested mixes achieved 100% vertical stability, surpassing their cement-paste counterparts (96–98%). In contrast, mixtures containing RCBW exhibited elevated water demand, reflected by a higher liquid-to-solid (L/S) ratio of 0.42; this effect was attributed to the high porosity and absorptivity of brick-derived wastes.

As highlighted by Robayo-Salazar et al. [19], achieving an optimal balance between workability and buildability remains a central challenge in 3DCP mix design. A higher L/S increases flowability and facilitates extrusion but degrades the dimensional stability of deposited layers; a lower L/S favors shape retention and interlayer cohesion yet limits flow, potentially disrupting process continuity. Rheological analyses have shown that adding GBW markedly increases the apparent viscosity of fresh mixtures—particularly under static conditions—by reducing free water and increasing interparticle friction, while simultaneously raising yield stress. As a result, resistance to deformation and the geometric stability of extruded layers increase, which is critical in 3DCP applications where the material must retain its form without formwork. It has been demonstrated that increasing the GBW content delays both the initial and final setting; for instance, a 10% GBW substitution extended the setting time by approximately 10 min. Importantly, the same mixture achieved the highest 28-day compressive strength, registering a 12.4% increase relative to the control mix when tested perpendicular to the printed layers [20]. In another study [21] mixtures with recycled brick aggregate (RBA) attained compressive strengths of 23.75 MPa (parallel to layer depositions, D1) and 29.06 MPa (perpendicular to layer depositions, D3), representing decreases of ~14% and ~20%, respectively, relative to a conventional reference material for 3DCP. The achieved D1/D3 ratio of 0.82 indicated anisotropic behavior and weakened interlayer bonding, a typical limitation of layer-to-layer processes resulting from suboptimal cohesion at the layer interfaces. Despite the observed reduction in mechanical parameters, these results confirm the technical feasibility of incorporating ceramic recyclates into 3DCP mixtures. Further advances, through optimization of aggregate properties, grading classification, surface modifications, or tailored admixture systems, may broaden the application window for recycled components in additive construction while mitigating trade-offs in structural performance.

Complementing the strategy of using non-reactive additions are lightweight, insulating fractions in 3D-printable mixes, such as expanded perlite, lightweight expanded clay aggregate (LECA), and EPS beads. Their inclusion reduces bulk density and thermal conductivity, improving the thermal performance of building envelopes while maintaining extrudability [22,23,24]. In practice, this necessitates control of particle grading and morphology to mitigate segregation during extrusion, pre-wetting of porous lightweight aggregates to stabilize rheology, reduce water uptake, and provide an additional “internal curing” effect, as well as compensation for strength reductions through optimization of the liquid-to-solid ratio, polycarboxylate ether (PCE) dosage, and fiber reinforcement [25,26]. As a spherical low-density filler, EPS can substantially lower thermal conductivity and density but, in the presence of insufficient matrix cohesion, may increase the risk of impaired interlayer bonding. Perlite and LECA typically improve buildability by raising apparent viscosity, yet they increase water absorption, which must be considered in admixture selection and curing. Consequently, properly engineered hybrid ‘structural–insulating’ mixtures can simultaneously satisfy printability requirements, extrudability, and shape stability, while enhancing the thermal performance of the wall [22,23,24].

Therefore, the present study investigates the physicochemical and performance-related properties of locally sourced waste materials and evaluates their possible applicability in 3DCP formulations. Mechanical properties, water absorption, and thermal conductivity of 3D-printed composites were determined. Particular focus was placed on potential recycled material use as non-reactive additions, both to stabilize the printing process and rheology, and to mitigate environmental impacts by reducing the consumption of natural aggregates and the landfilling of waste.

## 2. Materials and Methods

### 2.1. Materials

The reference mix was based on CEM I 52.5R Portland cement. The recipe was derived from the authors’ earlier work on 3DCP. Beyond meeting structural requirements, the mix needs to be designed to deliver the fresh-state properties essential for 3DCP: stable, continuous extrusion without clogging or segregation; dimensional stability after deposition (shape retention/buildability) sufficient to support subsequent layers without deformation or collapse; and high interlayer bond strength to ensure structural integrity and load transfer in the printed element. To meet these criteria, the composition was modified with a set of mineral additions and chemical admixtures. A set accelerator was used to shorten the time to early strength and stabilize freshly deposited layers. Rheology-controlling admixtures included a high-range water-reducing admixture (superplasticizer) and a viscosity-modifying agent (VMA) to balance flow with cohesion. Mineral fillers—most notably limestone powder and silica fume—were incorporated to tailor the cementitious matrix, increasing cohesion and effective surface area and thereby facilitating control over setting and hardening. The reference mix and all recycled additives were listed in Table 1.

#### 2.1.1. Origin and Processing of Recycled Feedstocks

Secondary constituents were sourced from typical construction and demolition (C&D) contexts, where substantial waste streams arise. While part of this material can be reused on-site, most requires logistics and processing to enable circular use. The re-use of 3DCP base material (printing waste) was taken into consideration. Due to its relative homogeneity and the absence of embedded steel reinforcement in current 3DCP practice, such waste can be readily reprocessed, which constitutes a significant advantage in terms of material circularity. Additional C&D fractions included mixed rubble, brick waste, and glass cullet. Mixed rubble is prevalent after full demolitions as well as partial renovations; effective reuse requires removal of rebar, wiring, plumbing elements, and other contaminants. Brick waste is more typical of low-rise and older residential construction, while glass waste originates from windows and glazing. Industrial by-products from the energy sector were also assessed, namely fly ash (FA) and ground granulated blast-furnace slag (GGBFS). The FA used originated from PGE Combined Heat and Power Plant (Kraków, Poland), and the slag from the Bogdanka power plant (Poland). All secondary additives were subjected to reprocessing, which involved crushing in a ball mill (Pulverisette 6, Fritsch GmbH, Idar-Oberstein, Germany) followed by sieving through a 4 mm mesh. Fly ash, collected as a fine powder, did not require crushing. To reduce density and improve thermal performance, three lightweight insulating fillers were used: expanded perlite (≤2 mm), fine lightweight expanded clay aggregate (LECA, ≤4 mm), and EPS beads (3–4 mm).

#### 2.1.2. Mix Compositions

The recycled constituents described above were incorporated at two replacement levels—20% and 40% by mass of the standard aggregate in the reference mix. For lightweight insulating fillers, three dosage levels were prepared to achieve progressively lower fresh densities; the highest dosage corresponded to the printability threshold for the equipment used. The full set of formulations investigated in this study is summarized in Table 1.

An additional set of formulations (Table 2) was developed by incorporating lightweight insulating fillers into the reference mixture, aiming to reduce the fresh density and partially replace the energy- and carbon-intensive reference material. These low-density mixtures are primarily intended for non-load-bearing applications (e.g., partition walls). Owing to their enhanced thermal performance, they may also be employed as infill in load-bearing wall systems, thereby providing an additional degree of thermal insulation.

### 2.2. Methods

#### 2.2.1. Particle Size Analysis

Particle size is critical to both rheology and processability in 3DCP. Grading, particle shape, and surface texture govern water demand as well as pumpability, extrudability, and buildability. Particle size distributions of the recycled fractions were measured using a laser-diffraction analyzer (Anton Paar PSA 1190D; Anton Paar GmbH, Graz, Austria) in accordance with ISO 13320:2020 [27]. Measurements were performed with wet dispersion in deionized water; sample preparation followed ISO 14887:2000 (Dispersing procedures for powders in liquids) [28]. The instrument’s built-in ultrasonic dispersion module was operated continuously to suppress agglomeration and maintain a stable, homogeneous suspension. The measurable size range was 0.5–3500 µm. All measurements were performed in triplicate to ensure repeatability and data reliability. Data acquisition and analysis were carried out using the manufacturer’s Kalliope software (version 2.22.1).

#### 2.2.2. Mixture Preparation

**Figure 1:**
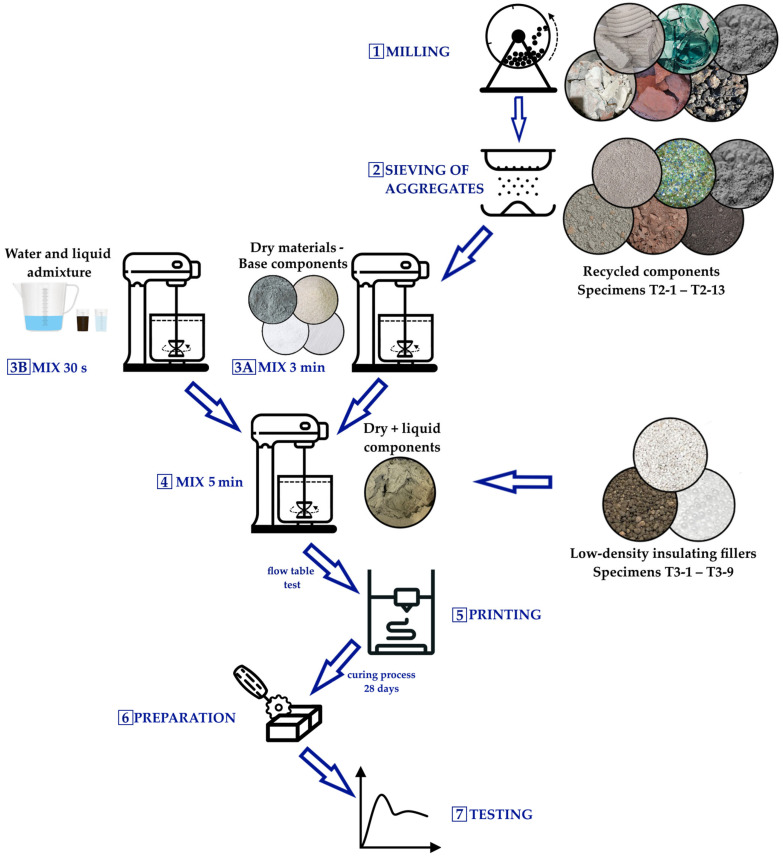
Schematic of the specimen preparation procedure for testing, covering steps 1 to 7.

Figure 1 outlines the workflow for recycled-feedstock reprocessing and mixture preparation. Recycled constituents were ground/milled for 6 h in a custom-built ball mill (charge volume ≈ 10 L) (Figure 1; Step 1). Following milling, the materials were sieved through a 4 mm sieve (Figure 1; Step 2), corresponding to the maximum particle size permitted by the laboratory-scale 3D printers used in this study, as constrained by screw and pump-based material transport mechanisms. Batching was performed on a precision laboratory scale (±0.1 g, capacity 20 kg). Mixing was carried out in a 20 L planetary mortar mixer (GEOLAB, Warsaw, Poland) following the protocol illustrated in Figure 1: dry (Figure 1; Step 3A) and liquid (Figure 1; Step 3B) constituents were premixed separately for 3 min and 30 s, respectively; the dry blend was then added to the liquid phase and mixed for an additional 5 min until a homogeneous mixture was obtained (Figure 1; Step 4). Prior to specimen fabrication, mixture consistency was verified by a flow-table test. Following consistency verification, specimens were printed using the 3D printer (Figure 1; Step 5). After 28 days of curing at 22 ± 2 °C, the printed elements were trimmed to the target dimensions (Figure 1; Step 6) and prepared for testing (Figure 1; Step 7).

#### 2.2.3. Consistency Verification

**Figure 2:**
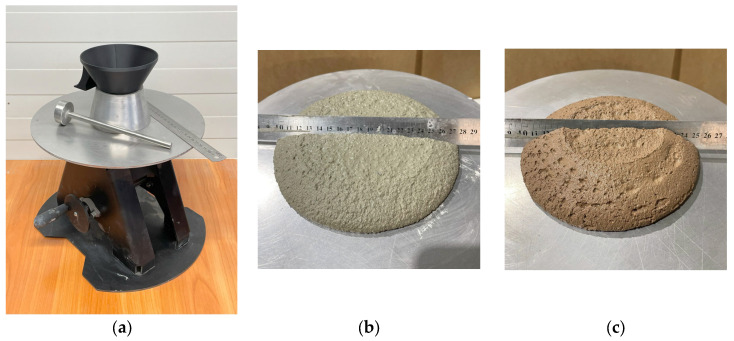
(**a**) View of the flow table test device according to the PN EN 1015-3 standard [29]; (**b**) material sample T2-9 at consistency test; (**c**) material sample T2-11 at consistency test.

Fresh-state consistency was assessed using the EN 1015-3 flow table test [29] (hand-operated apparatus; Figure 2). The standard truncated-cone mold was filled using a two-stage compaction procedure. After lifting the mold, the table was dropped 15 times. This method provides a small-sample indicator of printability by reflecting both extrudability and shape retention.

Proper consistency is essential for maintaining continuous material flow during printing and preserving the geometry of the printed element [30,31,32]. The screw-type extruder used in this study can process materials with an EN 1015-3 [29] flow spread of 120–200 mm, a range that ensures stable extrusion and adequate shape stability. In mixtures with recycled additions, a constant solid-to-water mass ratio (S/W) of 5:1 (*w*/*w*), equivalently, W/S = 0.20, yielded a reference flow spread of ~165 mm for specimen T2-1. This value provided stable extrusion and sufficient structural integrity after deposition. Other recycled mixes showed minor variations in flow spread that did not preclude printing; nonetheless, any deviation in consistency directly affects extrusion efficiency and may influence the geometry and surface quality of printed elements [31]. In mixtures containing lightweight insulating fillers, the S/W ratio was adjusted to account for the substantial change in mixture volume, which also affected consistency. At excessive dosages, reduced extrudability can become a limiting factor for printability.

#### 2.2.4. Printing and Preparation

**Figure 3:**
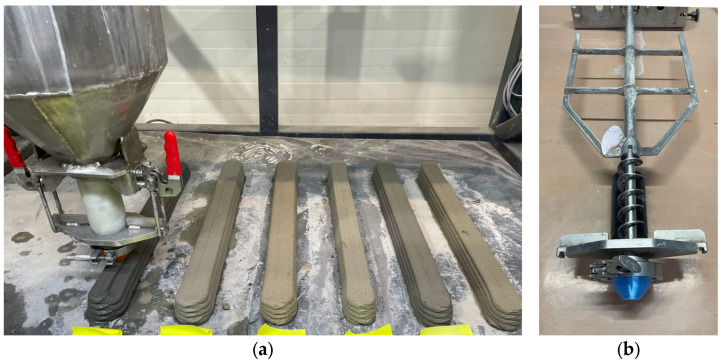

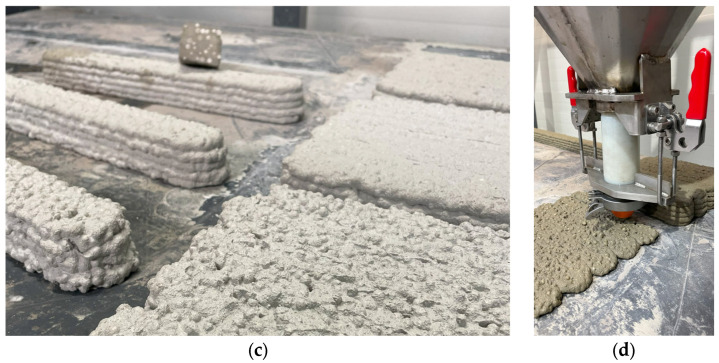
Laboratory setup for 3D concrete printing: (**a**) printer with printhead; (**b**) interior of the printhead with mixer and extruder; (**c**,**d**) example 3D-printed specimens with low-density components.

Specimens were produced on a laboratory 3DCP setup with a build volume of 1200 × 550 × 400 mm. The printer was equipped with a print head comprising a 20 L hopper with an integrated mixer and a screw-type extruder (Figure 3).

Following printing, the specimens were cured for 28 days under laboratory air-dry conditions, 23 °C and 40% RH, in accordance with practice for cementitious materials, to ensure adequate strength development.

#### 2.2.5. Flexural and Compressive Strength of 3DCP Samples

Because no dedicated standards exist for determining the mechanical properties of 3D-printed cementitious elements, investigation procedures were adapted from tests developed for cast materials.

A three-point bending test was conducted on the specimens to determine their flexural strength. The load was applied at a constant rate of 50 N/s until the specimen failed.

The following formula was used to calculate the flexural strength:

(1) $$\sigma_{max,\;B=}\frac{M_{g,max}}{W_{g}},$$

(2) $$M_{g,max}=\frac{F_{max}\cdot a}{4},$$

(3) $$W_{g}=\frac{bh^{2}}{6},$$

where

$\sigma_{max,\;B}$—maximum bending stress,(MPa);

$M_{g,\;max}$—maximum bending moment, (Nm);

$W_{g}$—section modulus for bending, (m^3^);

$F_{max}$—maximum failure load, $\left(kN\right);$

a—span between supports, (m);

b—width of the cross-section, (m);

h—height of the cross-section, (m).

Compressive strength tests in accordance with EN 196-1 [33] on an MTS Criterion 43 testing machine (MTS Systems, Eden Prairie, MN, USA), controlled with MTS TestSuite 1.0 software were performed by applying load to the specimens at a constant rate of 500 N/s until failure. Two testing configurations were investigated: (i) specimens with printed layers oriented perpendicular to the loading direction, and (ii) specimens with layers oriented parallel to the loading direction. In both cases, compressive strength was calculated using Equation (4):

(4) $$\sigma_{max,C=}\frac{F_{max}}{S}$$

where

$\sigma_{max,C}$—maximum stress in compression, compressive strength,(MPa);

$F_{max}$—load at failure, (N);

S—minimum cross-section determined for the narrowest cross-section, (${mm}^{2})$.

#### 2.2.6. Water Absorption Test

**Figure 4:**
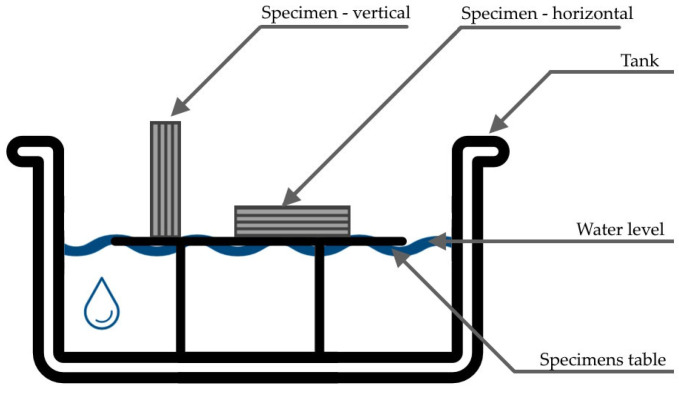
Schemes of water absorption test.

Water absorption was measured in a dedicated tank fitted with a plastic spacer (table) at the bottom to allow free water flow beneath the specimens. The water level was maintained at 3 mm above the support surface to ensure continuous but minimal contact, enabling controlled capillary uptake (Figure 4).

Additively manufactured specimens were tested in two orientations: (i) vertical, with a 40mm × 40 mm face in contact with water; and (ii) horizontal, with a 40 mm × 160 mm face in contact with water. The mass of each specimen was recorded at 1, 2, 4, 6, 12, 24, and 48 h from test start to quantify water uptake over time. In parallel, moisture ingress was monitored using a FLIR E96 thermal imaging camera; thermograms were captured every 5 min up to 4 h to visualize the wetting front.

The water absorption coefficient was determined using the following equation:

(5) $$A=\frac{∆m_{t}}{F\times ∆\sqrt{t}}\;,$$

where

*A*—water sorption coefficient, (kg/(m^2^h^1/2^));

$∆m_{t}$—sample mass increase, (kg);

*F*—suction surface, (m^2^);

$∆\sqrt{t}$—time square root increase, (h^1/2^).

#### 2.2.7. Thermal Conductivity

Thermal conductivity was measured with a FOX 314 heat-flow meter (LaserComp, New Castle, DE, USA; serial no. 1043). The instrument operates under steady-state conditions using heat-flux sensors and complies with:

- EN ISO 8301:1998 (guarded hot plate/heat-flow meter methods) [34].
- EN 12667:2002 (high/medium thermal resistance products; guarded hot plate/heat-flow meter) [35].
- ASTM C518-91 (heat-flow meter apparatus) [36].

**Figure 5:**
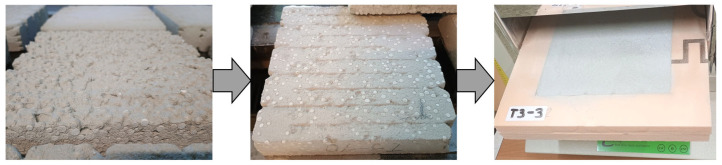
Schematic of specimen preparation for thermal conductivity testing of the 3D-printed material.

The maximum size of the measured sample can be 305 mm × 305 mm × 100 mm. For this study, plate-type specimens of 200 mm × 200 mm × 40 mm were 3D-printed. After 28 days of curing under controlled conditions, the prints were first cut to size to remove edge artifacts. Next, the top and bottom faces were ground to obtain smooth, plane surfaces as required. Each specimen was placed within an extruded polystyrene (XPS) frame to fill the test chamber and act as lateral insulation around the measurement zone. The specimen preparation scheme is shown in Figure 5.

Prior to testing, specimens were stored for 5 days under laboratory air-dry conditions at 23 °C and 40% RH.

#### 2.2.8. Microscopy Observation

Microscopic examination was performed using a Techrebal Banito B2920 digital microscope (Techrebal, Wilczyce, Poland) at 100× magnification, integrated with a Techrebal 48 MP camera (Techrebal, Wilczyce, Poland) for image acquisition.

## 3. Results and Discussion

### 3.1. Particle Size Analysis

Table 3 presents the particle-size distributions of the recycled aggregates used as additions. As is commonly known, particle size affects both water demand and hydration kinetics and thereby the resulting mechanical performance of the material [18,37].

Across all variants, the median size D_50_ was in the range of 15–26 µm. Among the investigated materials, fly ash (FA) and ground 3D-printed waste exhibit the narrowest distributions (smallest span) and lie predominantly in the fine (sub-63 µm) range. By contrast, glass cullet, mixed demolition rubble, and slag (GGBFS) display broader, coarser distributions (span ≈ 8.3–16.6) with substantial tails above 100 µm. For these types of materials, a more uniform grading would require longer milling and/or optimization of the ball charge (size and count) in the mill. The narrowest particle size distribution was recorded for FA. From a printability standpoint, narrow distributions with low D_90_, e.g., FA, 3D-printed waste, generally reduce water demand and yield more reproducible rheology [38], whereas broad, tail-heavy gradings (glass/mixed waste/GGBFS) tend to increase water demand and impair homogeneity. In 3DCP this may limit extrudability and raise the risk of nozzle clogging [39,40,41].

### 3.2. Mixture Consistency

**Figure 6:**
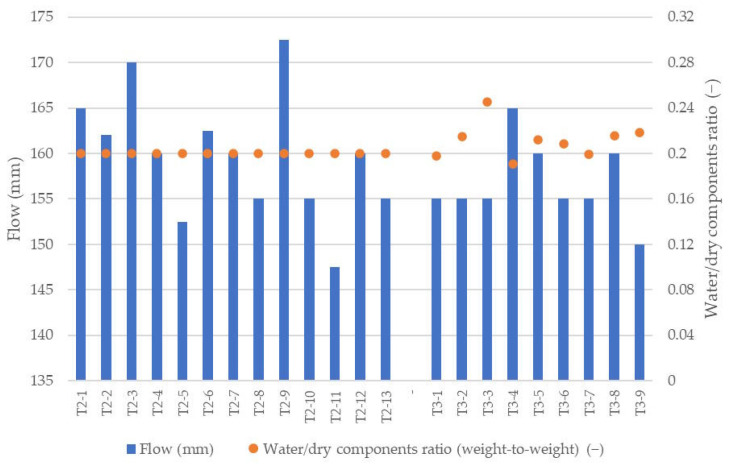
Consistency and water-solids ratio (W/S) of mixtures depending on their composition.

Consistency is critical in 3DCP, as it controls both extrusion continuity and shape stability of freshly deposited layers under the weight of subsequent layers. Flow-table results for all investigated mixes are shown in Figure 6.

The reference mix (T2-1) (no additions) achieved a flow spread of ≈165 mm, a desirable level that provided stable extrusion and sufficient post-deposition integrity. Nearly all recycled aggregates increased water demand relative to the reference mix, with the exception of glass (T2-3, T2-9), which produced a slightly higher spread (~170 mm). The largest decrease in spread occurred with ground brick (values < 150 mm), attributable to higher internal friction and water uptake associated with fine, angular brick fines [42,43,44]. FA and ground 3D-printed waste produced spreads closest to the reference. These variations did not materially disrupt printing; the only observable issue was a minor deformation in T2-9 (glass), linked to excess flowability and reduced layer stability, manifested as local settling during layer deposition.

For the lightweight series (T3-1 to T3-9), the water content was adjusted because the density change pushed consistency outside the printable window; spreads < 130 mm proved insufficient for extrusion. The adjustments and resulting spreads are summarized in Figure 6. The largest water increase was applied to the perlite mix (T3-3), raising *W/S* from 0.20 to 0.25 and yielding a spread of ≈155 mm.

### 3.3. Macro/Micro Observations of Specimens Cross-Sections

**Figure 7:**
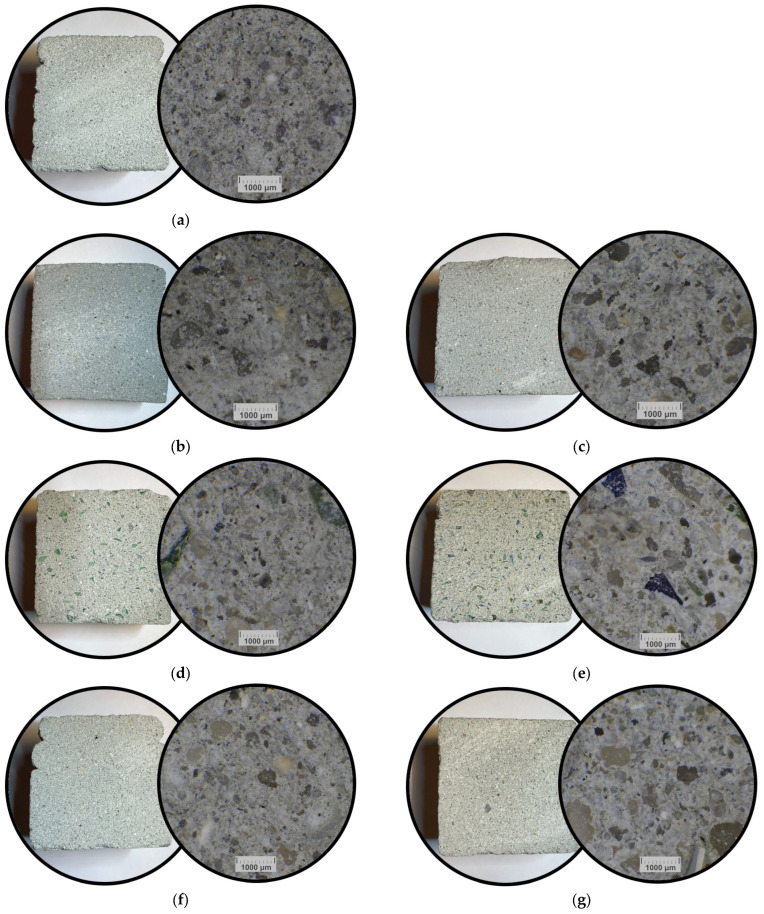

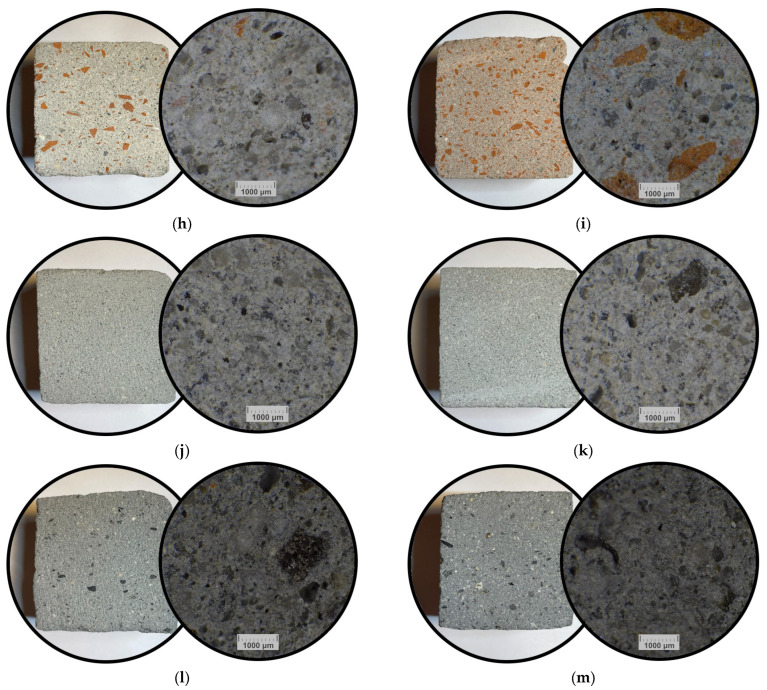
Representative view of cross-sections of specimens with recycled additives, left—macro photograph; right—micro photograph of: (**a**) T2-1; (**b**) T2-2; (**c**) T2-8; (**d**) T2-3; (**e**) T2-9; (**f**) T2-4; (**g**) T2-10; (**h**) T2-5; (**i**) T2-11; (**j**) T2-6; (**k**) T2-12; (**l**) T2-7; (**m**) T2-13.

All printed specimens were examined macroscopically and by optical microscopy on transverse sections. Macro images capture the distribution of coarse recycled constituents (e.g., glass, brick), while micrographs resolve the matrix-aggregate interfaces. Representative views for the 3DCP materials with recycled additives are compiled in Figure 7.

The reference specimen (T2-1) exhibits a homogeneous matrix with a dense microstructure. Visible small voids, most likely caused by particle pull-out during surface preparation. A similarly compact microstructure was observed for T2-6 (FA). At the higher FA dosage (T2-12), local heterogeneities become apparent in the micrograph. Other mixtures reveal a greater proportion of coarse particles (often >1 mm), most pronounced in the glass, brick, and slag series, which was consistent with observations from the macro-scale images. In all cases, the recycled constituents appear uniformly dispersed within the cementitious matrix. No interfacial cracking or debonding was observed at the contacts between the matrix and the coarse recycled grains. None of the recycled additions adversely affected printability. The specimens exhibited no visible cracks, voids, or defects at the layer interfaces, confirming appropriate printing parameters and adequate mixture consistency.

**Figure 8:**
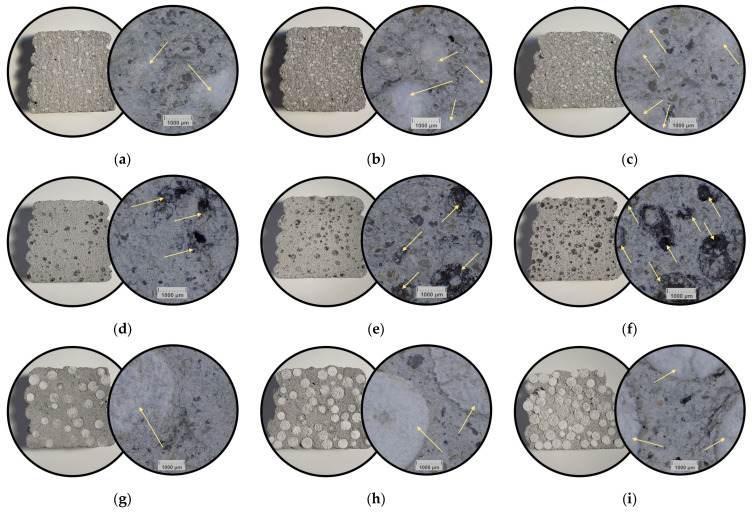
Representative view of cross-sections of specimens with recycled additives, left—macro photograph; right-micro photograph of: (**a**) T3-1; (**b**) T3-2; (**c**) T3-3; (**d**) T3-4; (**e**) T3-5; (**f**) T3-6; (**g**) T3-7; (**h**) T3-8; (**i**) T3-9. Arrows indicate the presence of a lightweight insulating additive.

An analogous microstructural analysis was performed for the lightweight formulations (Figure 8). Due to the strong color, texture contrast, and the volume fraction, the distribution of each lightweight filler was easy to observe.

In the case of specimens with insulating additions, the contrast in color between the additions and the reference material makes the effect of the addition content even more apparent. The finest addition in this set is expanded perlite, which is clearly visible when examining the macro images. The specimens produced with its inclusion, T3-1, T3-2, and T3-3, were characterized by a uniform distribution of this addition within the base matrix. The micrographs show perlite grains without sharply defined edges, which may result from the tendency of this addition to break up and abrade [45]. For perlite, it was important to add it in the final stage of mixing, yet early enough to distribute it uniformly throughout the base matrix, while avoiding excessively long mixing that would cause fragmentation [46,47].

LECA, in turn, exhibits rather clearly delineated particle edges; however, the internal structure of the granule was typically open, allowing the base matrix to penetrate its interior. Such cases are visible in the micrographs for specimens T3-5 and T3-6 (Figure 8e,f). This ingress of the base matrix into the particle interior may reduce the effectiveness of density reduction and limit the improvement in insulation. This is not a rule, however, since for specimen T3-4 the microstructural analysis indicates pores remaining inside the LECA grains.

The addition of granulated EPS was quite effective. At the highest content, the granulate fills almost the entire cross-section of the specimen. The structure was generally uniform, although there were locations where the EPS beads were in tight contact with one another and locations where a thicker coating of base matrix was visible around the bead. At lower dosages, the granulate was evenly dispersed. Microscopic analysis of the EPS-modified variants nevertheless reveals cracking at the phase boundary between the base matrix and the bead. These microcracks (particularly visible in Figure 8i) form during curing and result from the low adhesion of the granulate to the matrix. The hydrophobic, smooth EPS surface hinders wetting and bond formation. The modulus/compliance mismatch between the soft EPS and the matrix generates stress gradients at crack initiation sites. Such interfaces were regarded as a weak link and may directly and adversely affect the strength of elements printed with this material [48,49,50].

### 3.4. Water Absorption Test

**Figure 9:**
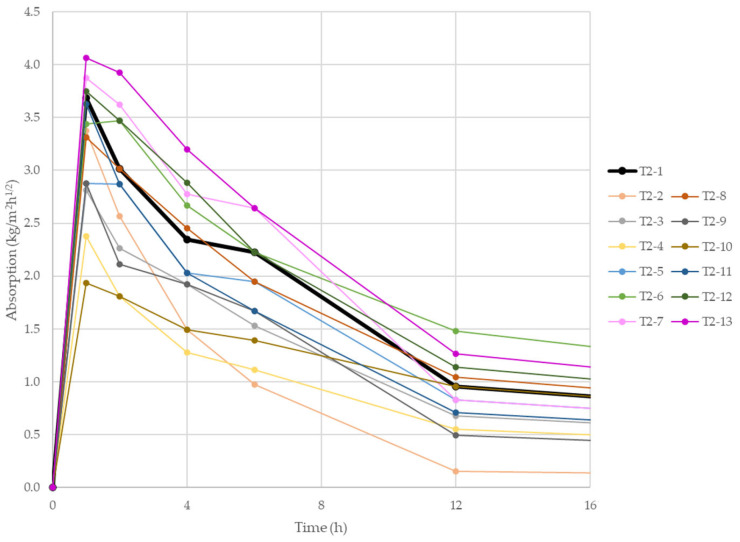
Absorption coefficient of 3D-printed specimens in vertical orientation depending on their composition.

**Figure 10:**
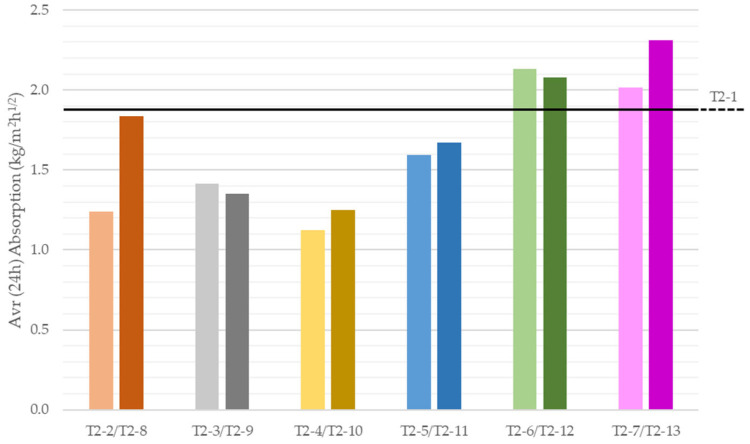
Average absorption coefficient of 3D-printed specimens in vertical orientation depending on their composition after 24 h of test.

Water absorption of the printed specimens as a function of mixture composition and printing direction was determined. Capillary uptake began immediately upon samples’ contact with the water surface. Most specimens absorbed water up to approximately half of their length. Horizontally oriented specimens appeared visibly wetter because their contact area with water was substantially larger, leading to greater uptake. Gravimetric mass tracking enabled calculation of the absorption coefficient. Figure 9 shows absorption-versus-time curves for vertically oriented specimens with recycled aggregates, and Figure 10 shows the average absorption coefficients after the first 24 h of test.

Figure 9 allows comparison of sorptivity across all recycled-aggregate mixes tested vertically. The most intense absorption occurred directly after test initiation: the 1 h average coefficient ranged from ~2 to ~4 kg/m^2^·h^1^ᐟ^2^. Subsequent readings declined progressively, falling to less than half by 12 h. Thereafter the rate diminished further and nearly ceased by ~48 h, as visible in the full test record (Figure S1, Supplementary Materials).

Specimens with mixed rubble additions (T2-4 and T2-10) exhibited the lowest 24 h average coefficients, 1.12 kg/m^2^·h^1^ᐟ^2^ (T2-4) and 1.25 kg/m^2^·h^1^ᐟ^2^ (T2-10). In this case, a higher recycled-aggregate content increased absorption; conversely, for glass and fly ash additions, higher dosages reduced absorption. The highest coefficients were observed for fly ash and slag mixes. Their 24 h averages value (T2-6, T2-7, T2-12, T2-13) exceeded 2 kg/m^2^·h^1^ᐟ^2^, surpassing the reference material (T2-1). This is somewhat unexpected given their particle-size distributions, especially FA, which showed the finest and most uniform grading, factors that would typically limit absorption [51,52]. Analogous trends were observed for horizontally tested specimens (Figures S2–S4, Supplementary Materials), albeit with slightly lower coefficients. This may relate to 3D printing’s layer-by-layer interfaces, which generally present increased porosity and reduced adhesion compared to, for example, cast monoliths. Such interfaces provide preferential fluid-migration pathways, promoting faster and more intense water ingress [52]. Moreover, printed microstructures are anisotropic: pores tend to align with the print direction, which facilitates capillary uptake-particularly along the layer orientation [53].

**Figure 11:**
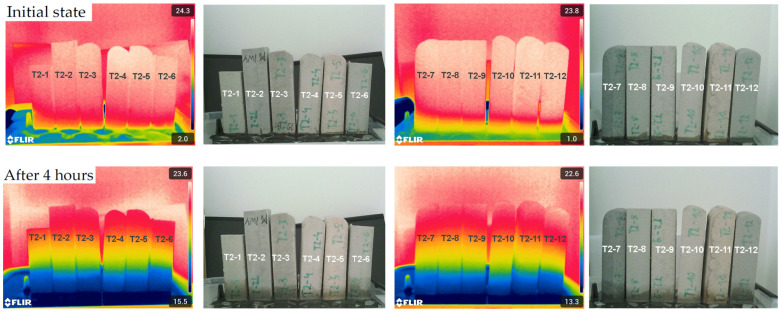
Images of the evolution of moisture propagation in samples numbered 1 to 12, recorded in both visible and infrared light, as a function of time and depending on the composition of the tested sample.

Infrared thermography confirmed the gravimetric results, although with lower discriminatory sensitivity, as the differences between specimens were less distinct (Figure 11). Across all series, absorption levels appeared broadly similar; slightly higher early-stage uptake was visible for T2-1 and T2-6 in the first comparison (Figure S5) and for T2-11 and T2 -12 in the second (Figure S6, Supplementary Materials).

**Figure 12:**
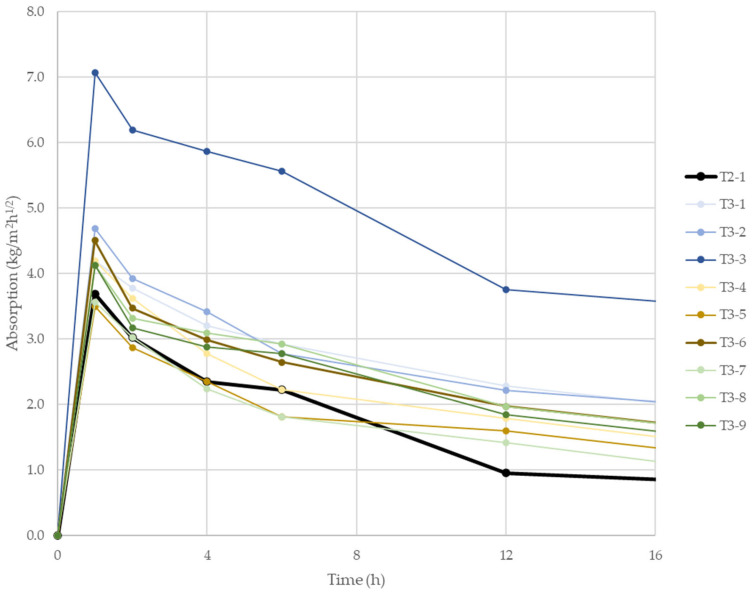
Absorption coefficient of 3D-printed specimens with low-density components in vertical orientation depending on their composition.

**Figure 13:**
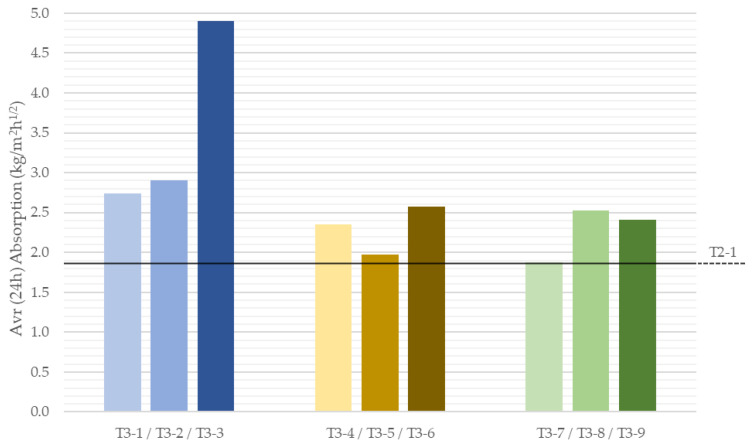
Average absorption coefficient of 3D-printed specimens with low-density components in vertical orientation depending on their composition after 24 h of test.

An analogous investigation was carried out for lightweight mixes that reduce fresh density. These tests were performed only in the vertical orientation. Figure 12 presents absorption-versus-time curves, and Figure 13 shows the average absorption coefficients after 24 h of test.

A similar overall pattern was observed: initially intense uptake followed by attenuation, yet for low-density mixes, the curve was less steep, i.e., water uptake persisted longer. This is evident in the late stage (Figure S7, Supplementary Materials), where after 48 h, the lightweight specimens were still absorbing, unlike the recycled-aggregate series. The 24 h average coefficients were also considerably higher (nearly all >2 kg/m^2^·h^1^ᐟ^2^) than those for recycled-aggregate mixes. Each of the analyzed variants was characterized by increased absorption compared to the reference sample (T2-1). The highest values occurred with expanded perlite, especially at the largest dosage, achieving a 24 h average of 4.89 kg/m^2^·h^1^ᐟ^2^. This may be linked to the particle attrition noted in the microscopy analysis for perlite, which can increase water uptake [46,47].

### 3.5. Bending Test

**Figure 14:**
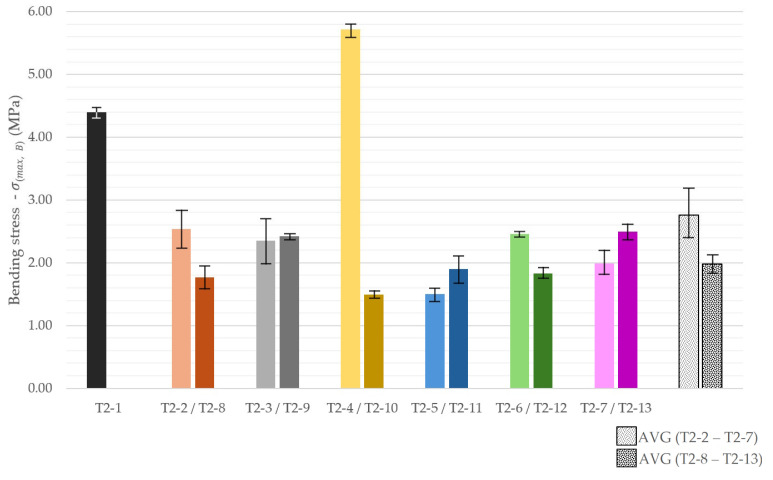
Flexural strength of 3D-printed concrete specimens depending on their composition.

Three-point bending showed that nearly all modifications relative to the reference mix decrease flexural strength (Figure 14). The sole exception was T2-4 (mixed rubble addition), which exceeded the value of reference material and reached 5.72 MPa. A plausible explanation of this phenomenon was local reinforcement due to residual fibers or other inclusions. Among the remaining variants, specimens with crushed 3D-printed waste, glass, fly ash, and slag (T2-2, T2-3, T2-6, T2-9, T2-13) clustered around 2–2.5 MPa, i.e., a little over 50% of the reference. The lowest results were recorded when the brick additions (T2-5, T2-11) were used. They reduced the flexural strength by approximately 60% compared to the reference mixture.

**Figure 15:**
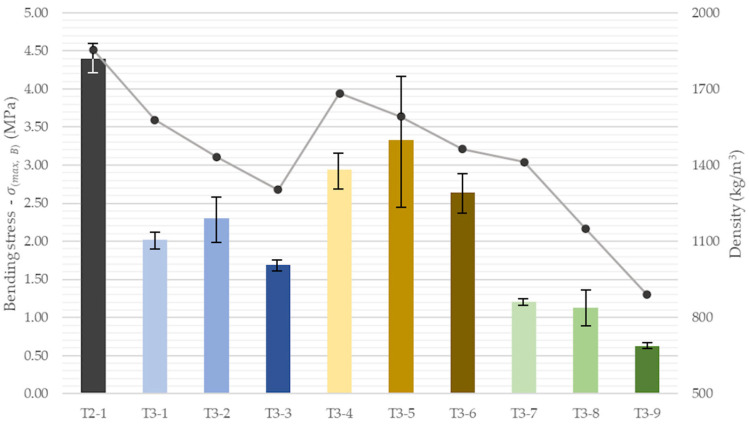
Flexural strength (bars) and density (line) of 3D-printed concrete specimens with insulation components.

Flexural strength of the lightweight series was analyzed in conjunction with the corresponding bulk density (Figure 15). The LECA mixes exhibited the smallest reductions relative to the reference material, averaging approximately 3 MPa (≈67% of the reference). Notably, the intermediate LECA dosage (T3-5) reached the highest mean value (3.33 MPa), albeit with considerable scatter. A similar trend was observed for the perlite mixes, where the intermediate variant (T3-2) displayed the highest strength (2.3 MPa). The lowest flexural strengths were recorded for the EPS mixes, averaging 0.99 MPa. Even the lowest-density EPS mix (T3-7, 1412 kg/m^3^) was approximately 28% weaker than the corresponding perlite mix T3-3 (1303 kg/m^3^). This reduction in strength is consistent with the microstructural observations of matrix-EPS interfacial microcracking reported in Section 3.3, which contribute to weakening of the printed elements.

### 3.6. Compressive Test

**Figure 16:**
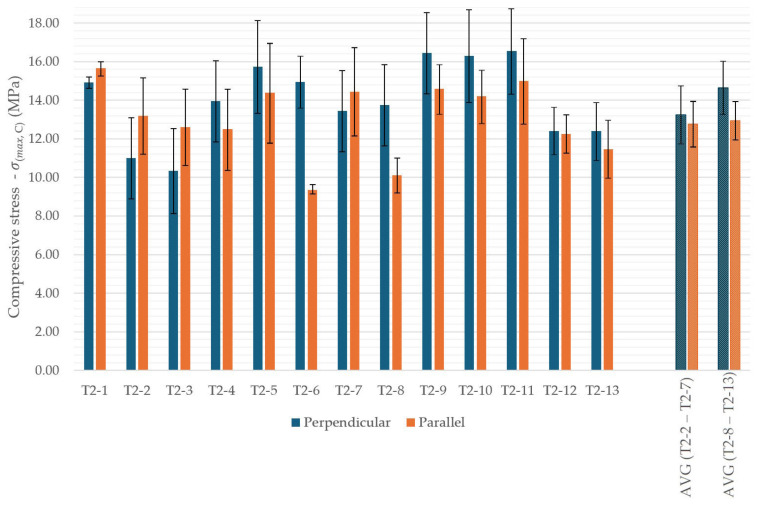
Compressive strength of 3D-printed concrete specimens with recycled components, depending on specimen orientation.

**Figure 17:**
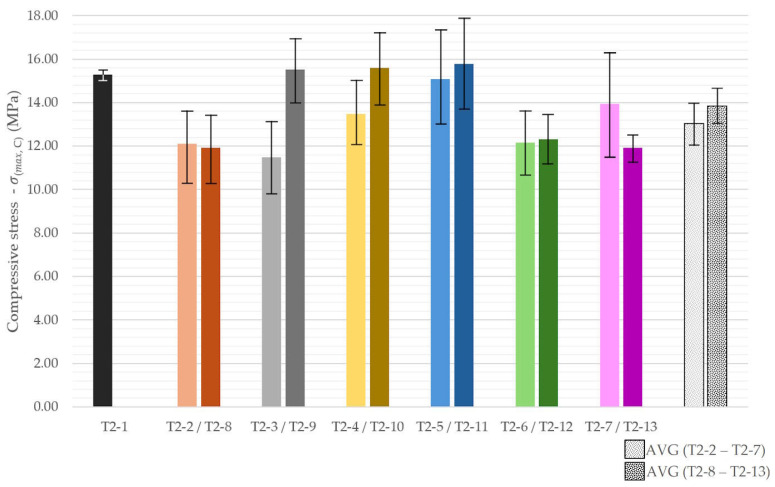
Compressive strength of 3D-printed concrete specimens with recycled components.

Compressive strength results were presented in Figure 16. Moreover, Figure 17 shows the averaged strength values for each material variant.

For the recycled-aggregate mixtures, anisotropy inherent to layer-wise fabrication was observed. In the lower-replacement group (T2-2 to T2-7), loading perpendicular to the printed layers resulted in compressive strengths approximately 4% higher than loading parallel to the layers. In the higher-replacement group (T2-8 to T2-13), this difference increased to roughly 13%. The reference mix exhibited comparable strengths in both orientations, whereas the largest orientation-dependent effects were recorded for T2-6 and T2-8.

In terms of absolute strength, specimens T2-9, T2-10, and T2-11 exceeded the reference material, reaching 15.5–16.0 MPa. These mixtures incorporated aggregates with a broad particle-size distribution, which, while slightly detrimental for flexural performance, appeared beneficial for compressive strength due to improved particle packing. Contrary to the flexural trends, the higher-replacement group (T2-8 to T2-13) achieved, on average, approximately 6% higher compressive strength than the lower-replacement group. The lowest compressive strengths were observed in mixtures containing crushed 3D-printed waste.

**Figure 18:**
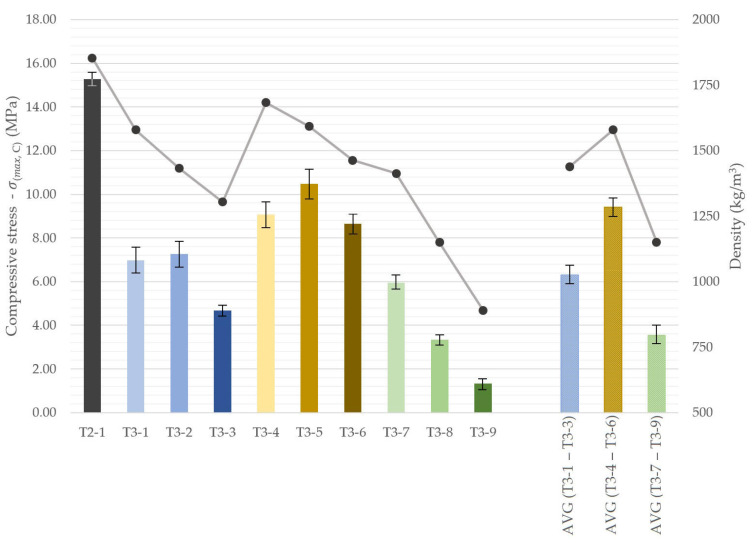
Compressive strength (bars) and density (line) of 3D-printed concrete specimens with insulation components, depending on specimen orientation.

**Figure 19:**
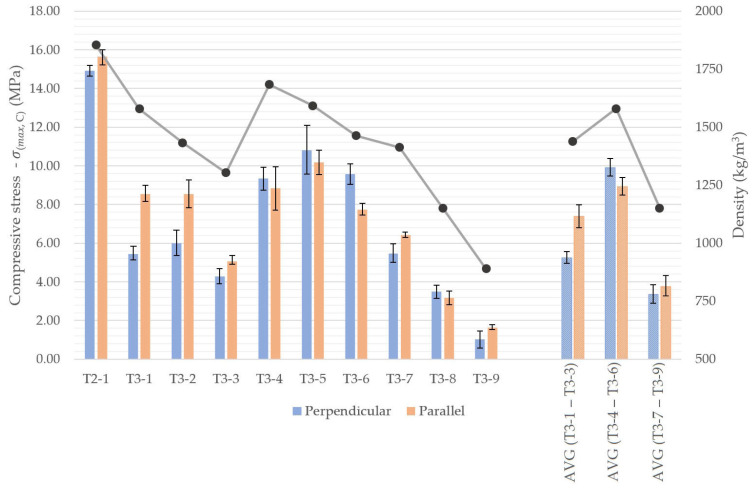
Compressive strength (bars) and density (line) of 3D-printed concrete specimens with recycled components.

For the lightweight series, the compressive tests revealed orientation-dependent trends presented in Figure 18, with the corresponding average values summarized in Figure 19. In these mixtures containing insulating fillers, the effect of printing orientation was more dependent on the specific formulation.

Perlite specimens (T3-1 to T3-3) exhibited higher flexural strength when loaded parallel to the printed layers, with increases of up to 40%. EPS mixes (T3-7 to T3-9) showed a similar trend, albeit with a smaller difference of approximately 12%. In contrast, LECA specimens demonstrated higher strength when loaded perpendicular to the printed layers, with an average value of 9.9 MPa.

In terms of absolute values, the LECA mixes (T3-4 to T3-6) exhibited the highest compressive strengths, averaging 9.41 MPa, in agreement with the flexural performance trends. This behavior is consistent with density effects, as LECA reduced the bulk density the least, by an average of 14.7% relative to the reference mix. In contrast, EPS, although most effective in reducing density, resulted in the lowest compressive strengths, averaging below 4 MPa, corresponding to approximately 23% of the reference material.

### 3.7. Thermal Conductivity and Density

**Figure 20:**
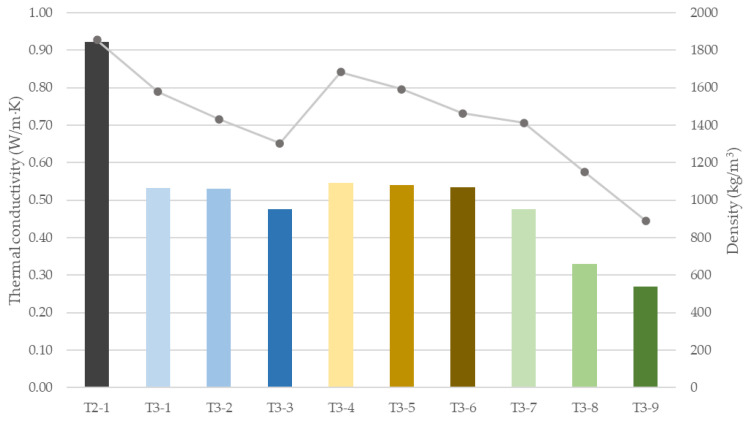
Thermal conductivity (λ, bars) and density (line) of 3D-printed specimens with lightweight insulating additions.

Figure 20 shows the thermal conductivity λ and density of all investigated samples.

Figure 20 reveals a clear dependency between density and thermal conductivity. As mixture density decreases, thermal conductivity is reduced, although the magnitude of this effect is strongly dependent on the type of lightweight addition. Expanded perlite (T3-1 to T3-3) reduced fresh density by 15–30% compared with the reference mix, resulting in a marked decrease in conductivity of up to 48.4%, with a minimum value of 0.475 W/m·K for T3-3. For perlite, the density–conductivity relationship is the least linear (R^2^ = 0.75). Lightweight expanded clay aggregate (LECA) exhibited the least pronounced response: density reductions of 9–21% yielded a minimum conductivity of 0.535 W/m·K at the highest dosage (T3-6), corresponding to a ~41% decrease relative to the reference. As anticipated, EPS beads were the most effective, lowering density by 24–52% (down to 890 kg/m^3^ for T3-9) and producing the lowest conductivity of 0.271 W/m·K, equivalent to a ~71% reduction versus the reference. For both EPS and LECA, the conductivity–density trend was close to linear (R^2^ = 0.95 and R^2^ = 0.97, respectively). These findings demonstrate that all three lightweight additions improve thermal performance, with EPS providing the strongest insulating effect, perlite delivering a substantial intermediate benefit, and LECA offering a more moderate reduction in λ, consistent with its smaller impact on density.

**Figure 21:**
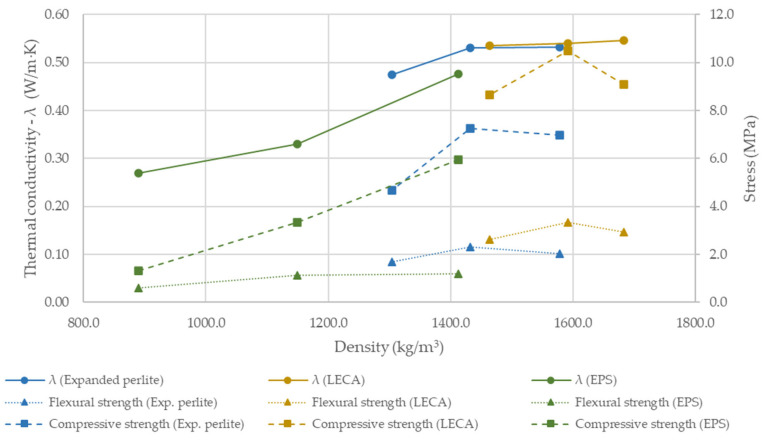
Thermal conductivity (λ), flexural strength, and compressive strength as functions of material density and the insulating additive used.

However, when applying the multi-criteria analysis (Figure 21), it becomes apparent that, despite thermal performance comparable to perlite, LECA attains substantially higher compressive and flexural strengths (by nearly 50% in both metrics). The EPS-based mix, while the most effective insulator, incurs the greatest losses in mechanical strength. Accordingly, its use would be confined to non-load-bearing applications or to insulating infill layers that markedly enhance the thermal performance of building envelopes. In terms of jointly maximizing mechanical properties and improving thermal insulation, LECA emerges as an effective compromise.

## 4. Conclusions

Three-dimensional concrete printing (3DCP) is developing rapidly; however, its implementation should be coupled with sustainability strategies consistent with circular-economy principles. The present study assessed the feasibility of substituting natural aggregates with recycled constituents and incorporating lightweight insulating fillers to reduce density-and thereby the embodied energy and carbon—of printable cementitious mixtures.

Recycled aggregates can effectively reduce natural-aggregate demand provided that grading and surface quality are controlled. Narrow particle-size distributions (e.g., FA, ground 3DCP waste) were favorable, whereas broad gradings (glass, mixed rubble, slag) increased water demand and extrusion risks. Optimizing milling conditions and grading curves is essential.

Printability remained within acceptable limits regardless of the composition of the tested mixtures. Flow spread decreased slightly with most recycled additions (brick being the lowest), while glass increased it. Printing stability was generally preserved.

Flexural strength tended to decrease with recycled constituents, though isolated improvements were observed (e.g., rubble mix T2-4). Lower replacement levels generally allowed for better results.

Compressive strength benefited from broader gradings in certain mixes and displayed the anisotropy typical of 3DCP. Higher replacement levels yielded ~6% strength gains due to improved packing, with perpendicular loading showing up to 13% higher values than parallel loading.

Lightweight fillers improved thermal performance at the expense of mechanical strength. LECA provided the most favorable compromise, perlite showed intermediate behavior, and EPS offered the strongest insulation but caused significant strength losses due to weak matrix-EPS interfaces.

Water absorption decreased substantially in recycled-aggregate mixes after 48 h, while lightweight mixes exhibited more persistent uptake, especially at high perlite dosages.

The obtained results demonstrate that non-reactive recycled constituents and lightweight insulating fillers can be successfully employed in 3DCP to reduce environmental impact while maintaining printability. Optimized particle-size distribution, orientation-aware structural design, and improved matrix–filler interfaces are key to achieving target performance, particularly in EPS-modified systems.

## Figures and Tables

**Table 1:** Composition and sample designation of the investigated mixtures incorporating recycled additives.

Specimens Designation	Base Components			Recycled Components					
	Cement CEM I	Quartz Sand	Additions	3D-Printed Waste	Glass	Mixed Waste	Brick	FA	GGBFS
	**(%)**								
T2-1	25	65	10	-	-	-	-	-	-
T2-2	25	45	10	20	-	-	-	-	-
T2-3	25	45	10	-	20	-	-	-	-
T2-4	25	45	10	-	-	20	-	-	-
T2-5	25	45	10	-	-	-	20	-	-
T2-6	25	45	10	-	-	-	-	20	-
T2-7	25	45	10	-	-	-	-	-	20
T2-8	25	25	10	40	-	-	-	-	-
T2-9	25	25	10	-	40	-	-	-	-
T2-10	25	25	10	-	-	40	-	-	-
T2-11	25	25	10	-	-	-	40	-	-
T2-12	25	25	10	-	-	-	-	40	-
T2-13	25	25	10	-	-	-	-	-	40

**Table 2:** Composition and sample designation of the investigated mixtures with insulation additives.

Specimens Designation	Base Components			Low-Density Insulating Fillers		
	Cement CEM I	Quartz Sand	Additions	Expanded Perlite	Expanded Clay (LECA)	EPS
	**(%)**					
T3-1	24.58	64.58	9.58	1.25	-	-
T3-2	24.17	64.17	9.17	2.5	-	-
T3-3	23.75	63.75	8.75	3.75	-	-
T3-4	23.42	63.42	8.42	-	4.75	-
T3-5	21.83	61.83	6.83	-	9.5	-
T3-6	19.47	59.47	4.47	-	16.6	-
T3-7	24.92	64.92	9.92	-	-	0.25
T3-8	24.83	64.83	9.83	-	-	0.5
T3-9	24.75	64.75	9.75	-	-	0.75

**Table 3:** Particle size distribution of recycled aggregate after milling and sieving used as an additive to concrete mixtures.

Samples Designation	D_10_ (μm)	D_50_ (μm)	D_90_ (μm)	Mean Size (μm)	Span (D_90_-D_10_)/D_50_
3D-Printed elem.	1.99	16.29	80.08	31.98	4.79
Glass	1.88	20.25	294.11	92.16	14.43
Mixed waste	1.67	16.35	273.68	85.81	16.64
Brick	1.31	15.89	173.35	29.76	10.83
Fly Ash (FA)	2.98	25.28	72.84	34.06	2.76
Slag (GGBFS)	2.95	25.72	216.89	48.75	8.32

## Data Availability

The original contributions presented in this study are included in the article and Supplementary Materials. Further inquiries can be directed to the corresponding author.

## Supplementary Materials

The following supporting information can be downloaded at https://www.mdpi.com/article/10.3390/ma18184387/s1. Figure S1. Absorption coefficient of 3D printed specimens with recycled components in vertical positions—test 0–48 h.; Figure S2. Absorption coefficient of 3D printed specimens with recycled components in horizontal positions.; Figure S3. Absorption coefficient of 3D printed specimens with recycled components in horizontal positions—test 0–48 h.; Figure S4. Average absorption coefficient from 24 h of 3D printed specimens with recycled components in vertical positions.; Figure S5. Images of the evolution of moisture dispersion in samples numbered 1 to 6, recorded in both visible and infrared light, as a function of time and depending on the composition of the tested sample.; Figure S6. Images of the evolution of moisture dispersion in samples numbered 7 to 12, recorded in both visible and infrared light, as a function of time and depending on the composition of the tested sample.; Figure S7. Absorption coefficient of 3D printed specimens with insulation components in hori-zontal positions—test 0–48 h.; Table S1: Summary one-way analysis of variance (ANOVA) of compressive strength results.; Table S2: Two-sample *t*-tests (equal variances assumed) between T2-1 and other groups.

## Abbreviations

The following abbreviations are used in this manuscript:

3DCP	3D Concrete Printing
C&D	Construction and demolition
CDW	Construction and demolition waste
CEM I	Portland cement, Type I
D50	Median particle size (50th percentile)
D90	90th percentile particle size
EN	European Standard
EPS	Expanded polystyrene
EU-27	European Union (27 Member States)
FA	Fly ash
GBW	Ground brick waste
GGBFS	Ground granulated blast-furnace slag
ISO	International Organization for Standardization
LECA	Lightweight expanded clay aggregate
L/S	Liquid-to-solid ratio (by mass)
OPC	Ordinary Portland cement
PCE	Polycarboxylate ether (superplasticizer)
PGE	Polska Grupa Energetyczna (Polish Energy Group)
RBA	Recycled brick aggregate
RCA	Recycled concrete aggregate
RCBW	Recycled clay brick waste
RFA	Recycled fine aggregate
RH	Relative humidity
SBU	Structural build-up
S/W	Solids-to-water ratio (by mass)
VMA	Viscosity-modifying agent
W/S	Water-to-solids ratio (by mass)
XPS	Extruded polystyrene
