# Supplementary material for: Recycled Materials and Lightweight Insulating Additions to Mixtures for 3D Concrete Printing
Source: Materials (Basel). 2025 Sep 19;18(18):4387. doi: 10.3390/ma18184387 (PMC12471513; doi:10.3390/ma18184387)
Supplement: Supplementary file 1 [file materials-18-04387-s001.zip › materials-3877620-supplementary.pdf]

# Supplementary Materials

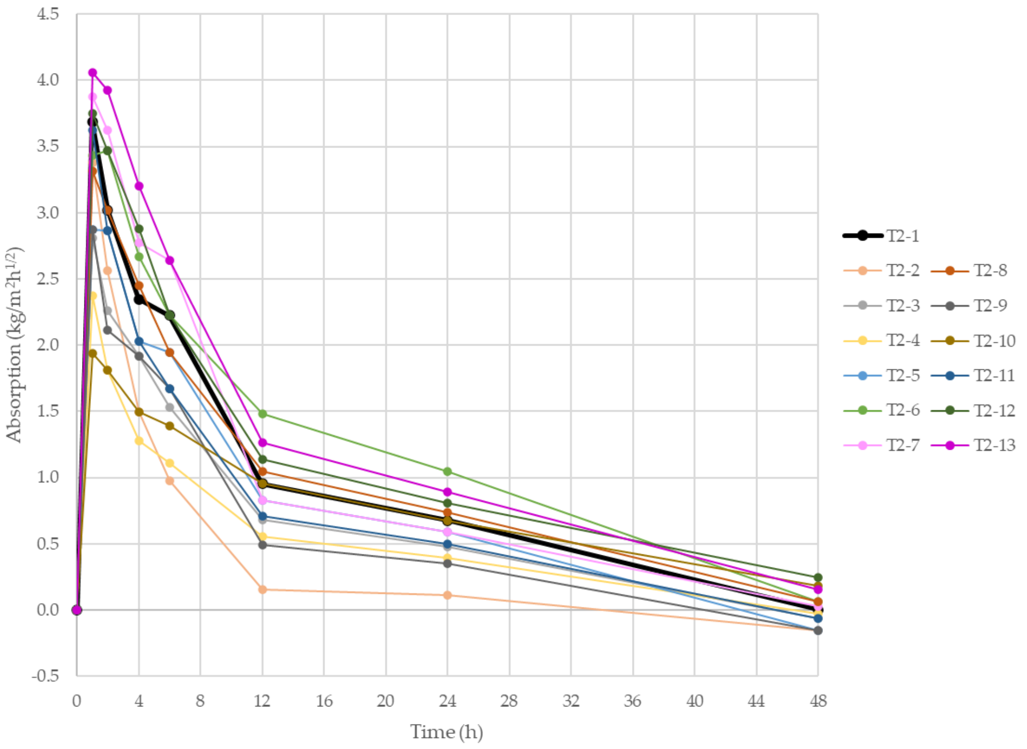


**Figure S1.** Absorption coefficient of 3D printed specimens with recycled components in vertical positions – test 0-48 h.

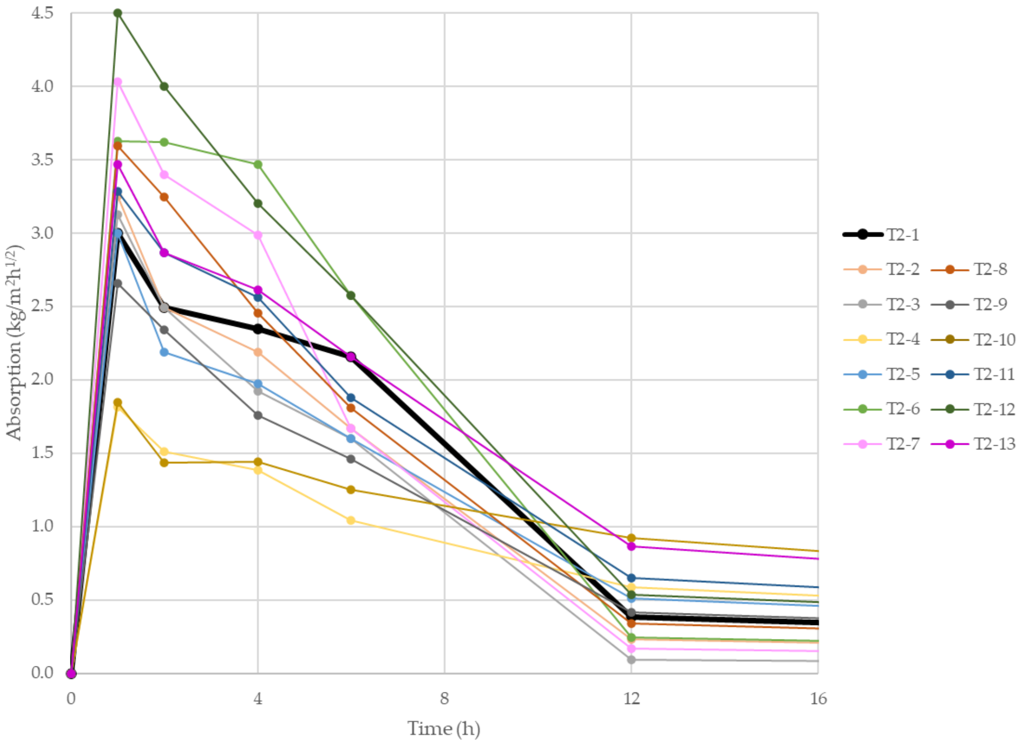


**Figure S2.** Absorption coefficient of 3D printed specimens with recycled components in horizontal positions.

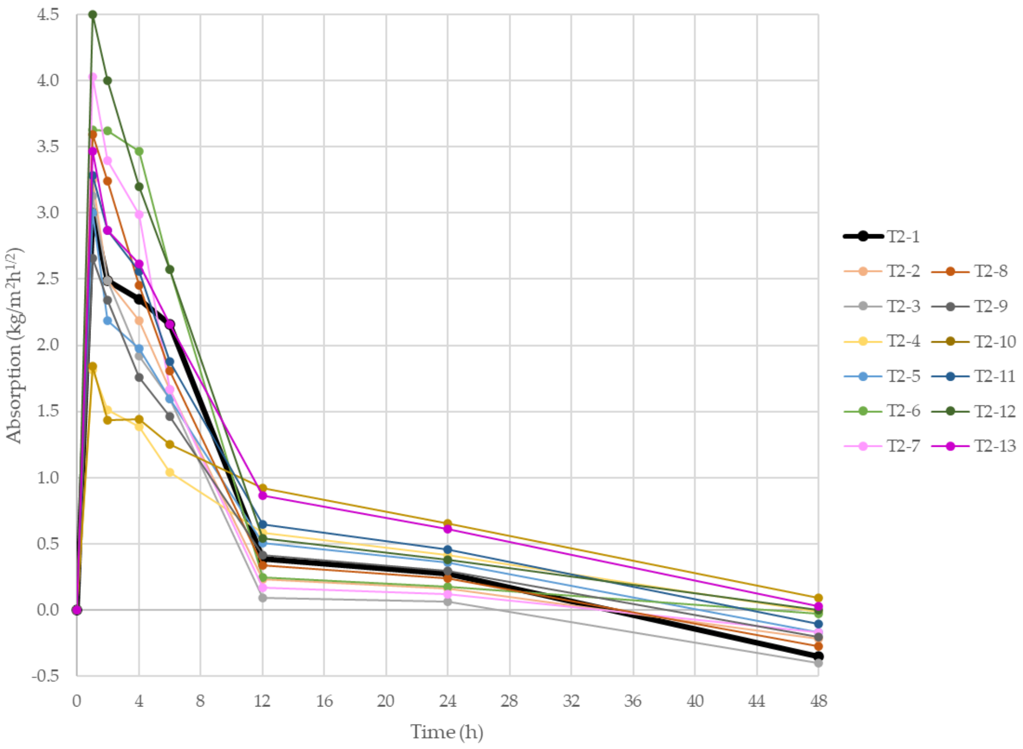


**Figure S3.** Absorption coefficient of 3D printed specimens with recycled components in horizontal positions – test 0-48 h.

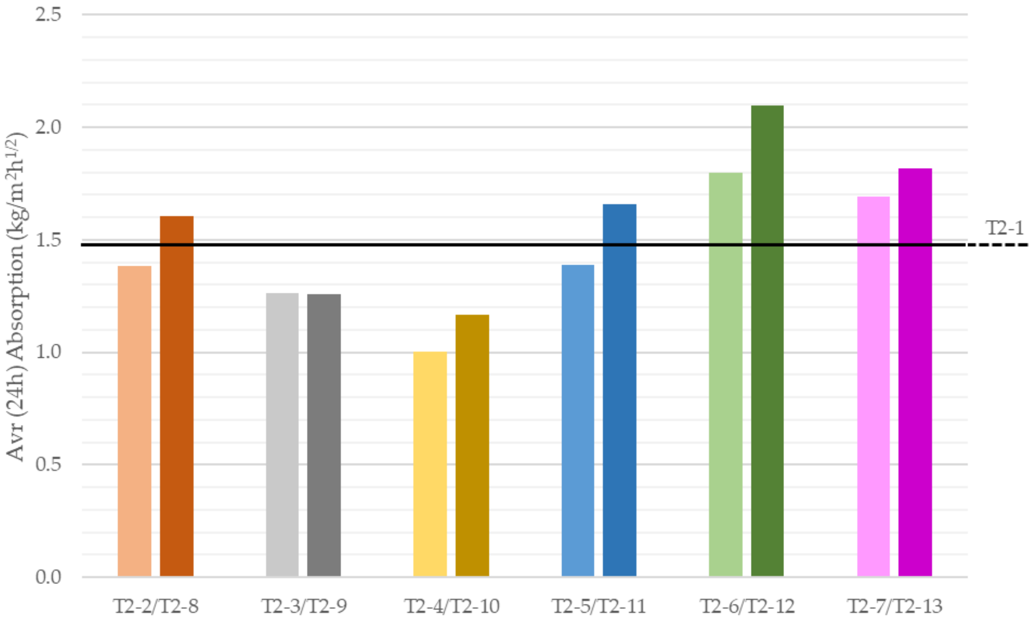


**Figure S4.** Average absorption coefficient from 24 hours of 3D printed specimens with recycled components in vertical positions.

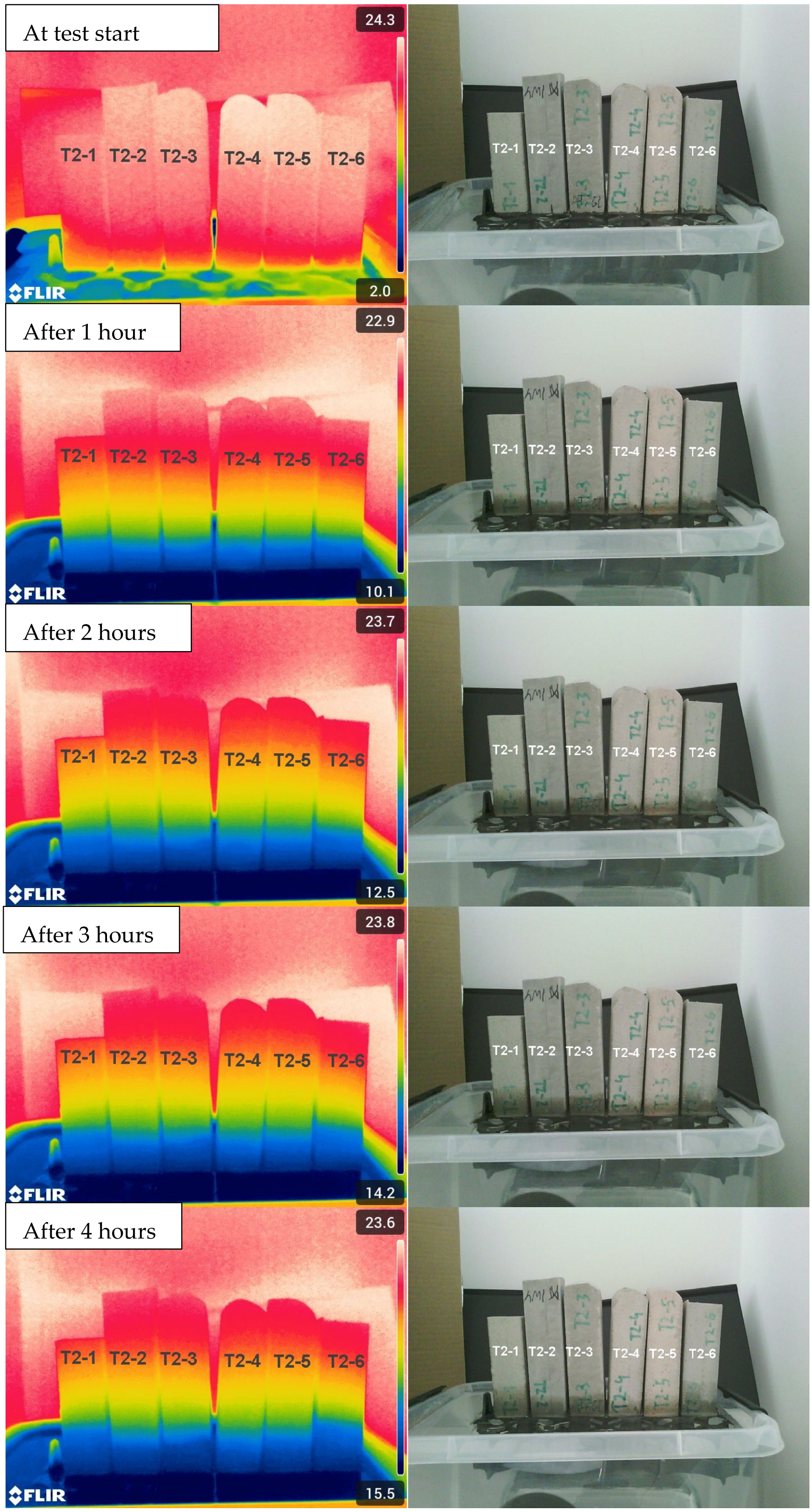


**Figure S5.** Images of the evolution of moisture dispersion in samples numbered 1 to 6, recorded in both visible and infrared light, as a function of time and depending on the composition of the tested sample.

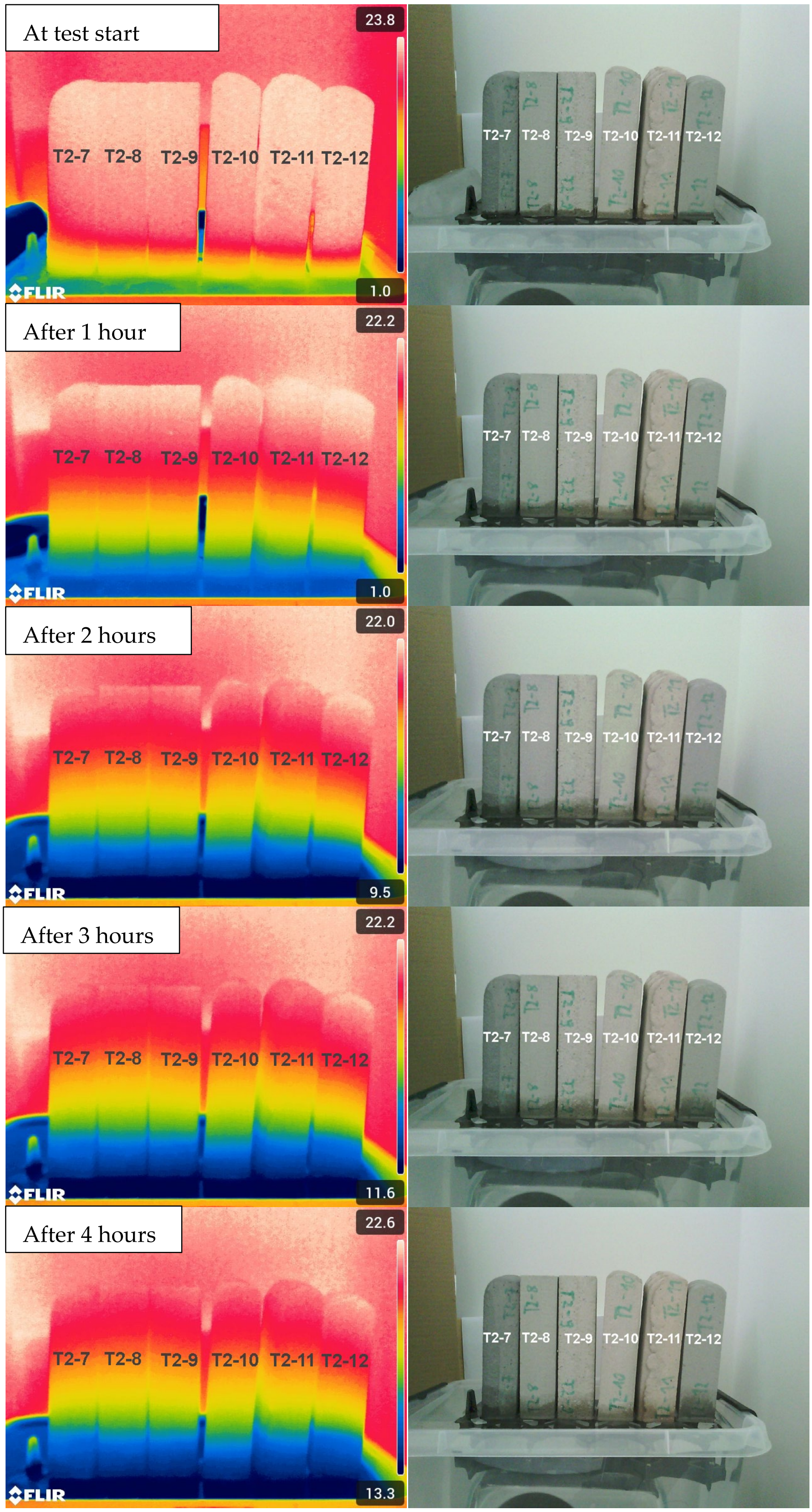


**Figure S6.** Images of the evolution of moisture dispersion in samples numbered 7 to 12, recorded in both visible and infrared light, as a function of time and depending on the composition of the tested sample.

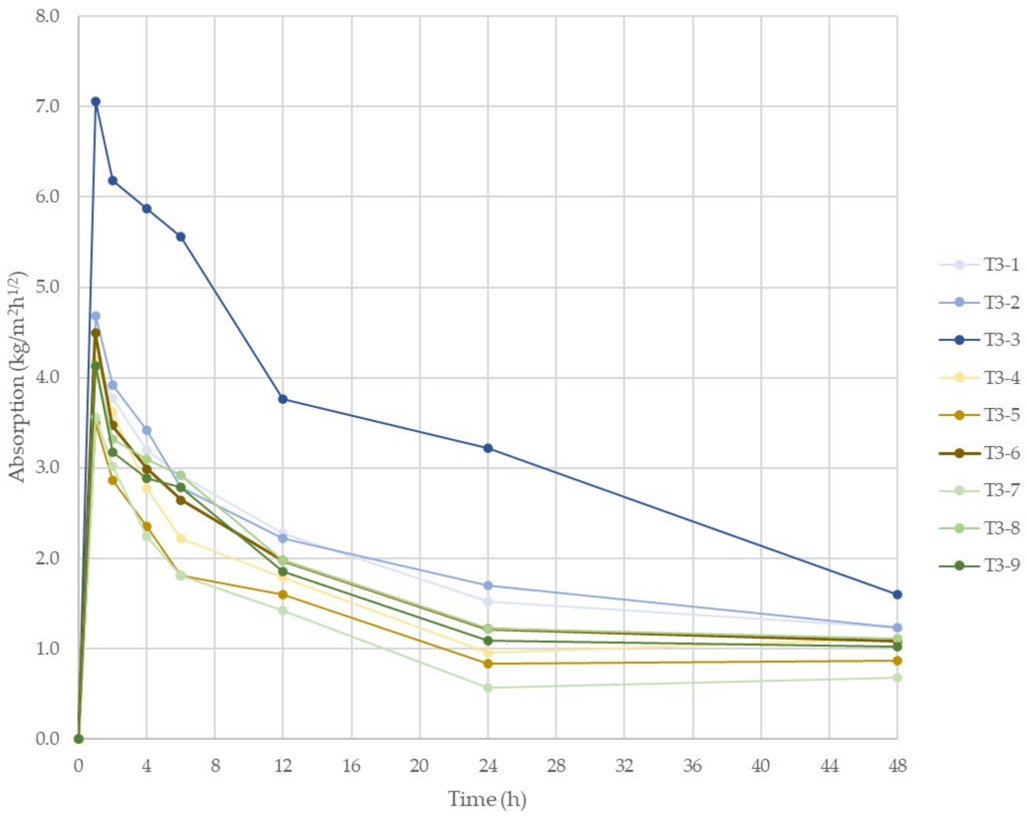


**Figure S7.** Absorption coefficient of 3D printed specimens with insulation components in horizontal positions – test 0-48 h.

Table S1. Summary one-way analysis of variance (ANOVA) of compressive strength results.

| Specimens designation | *n* | *Average* | *Variance* |
|---|---|---|---|
| T2-1 | 4 | 15.3 | 0.3 |
| T2-2 | 4 | 12.1 | 16.1 |
| T2-3 | 4 | 11.5 | 19.4 |
| T2-4 | 4 | 13.5 | 10.3 |
| T2-5 | 4 | 15.1 | 32.7 |
| T2-6 | 4 | 12.2 | 14.5 |
| T2-7 | 4 | 14.0 | 45.7 |
| T2-8 | 4 | 11.9 | 15.8 |
| T2-9 | 4 | 15.5 | 15.1 |
| T2-10 | 4 | 15.6 | 11.6 |
| T2-11 | 4 | 15.8 | 36.5 |
| T2-12 | 4 | 12.3 | 11.0 |
| T2-13 | 4 | 11.9 | 2.5 |

A one-way analysis of variance (ANOVA) conducted for thirteen groups (T2-1 – T2-13) did not reveal statistically significant differences between the group means ($F = 0.63$, $p = 0.80$). The obtained results indicate that the observed differences between groups were small compared to the within-group variability.

Pairwise comparisons between group T2-1 and the remaining groups (T2-2 – T2-13) were performed using a two-sample t-test assuming equal variances.

Table S2. Two-sample t-tests (equal variances assumed) between T2-1 and other groups.

| | *T2-1* | Significance |
|---|---|---|
| T2-2 | 0.0844 | ns |
| T2-3 | 0.0692 | ns |
| T2-4 | 0.1565 | ns |
| T2-5 | 0.4734 | ns |
| T2-6 | 0.0777 | ns |
| T2-7 | 0.3549 | ns |
| T2-8 | 0.0732 | ns |
| T2-9 | 0.4514 | ns |
| T2-10 | 0.4286 | ns |
| T2-11 | 0.4373 | ns |
| T2-12 | 0.0655 | ns (trend) |
| T2-13 | 0.0037 | *** |

ns – not significant; * $p \leq 0.05$; ** $p \leq 0.01$, *** $p \leq 0.005$

Significant differences were observed only for the comparison between T2-1 and T2-13 ($p = 0.0037$), indicating that the mean value of T2-13 was statistically lower than that of T2-1. All other comparisons yielded non-significant results ($p > 0.05$).
